# Diagnosis of Transverse Sinus Hypoplasia in Magnetic Resonance Venography: New Insights Based on Magnetic Resonance Imaging in Combined Dataset of Venous Outflow Impairment Case–Control Studies: Post Hoc Case–Control Study

**DOI:** 10.1097/MD.0000000000002862

**Published:** 2016-03-11

**Authors:** Ke Han, A-Ching Chao, Feng-Chi Chang, Hung-Yi Hsu, Chih-Ping Chung, Wen-Yung Sheng, Lung Chan, Jiang Wu, Han-Hwa Hu

**Affiliations:** From the Department of Neurology and Neuroscience Center, First Hospital of Jilin University, Changchun, Jilin, China (KH, JW), Department of Neurology, College of Medicine, Kaohsiung Medical University and Department of Neurology, Kaohsiung Medical University Hospital, Kaohsiung (A-CC), Department of Radiology (F-CC), Department of Neurology Veterans General Hospital and National Yang-Ming University (C-PC, W-YS), Department of Neurology, Tungs’ Taichung Metro Harbor Hospital and Department of Neurology, School of Medicine, Chung Shan Medical University, Taichung (H-YH), Department of Neurology, Taipei Medical University-Shaung Ho Hospital (LC), and Graduate Institute of Clinical Medicine and Department of Neurology, College of Medicine, Taipei Medical University and Hospital (H-HH), Taipei, Taiwan.

## Abstract

In previous studies of transverse sinus (TS) hypoplasia, discrepancies between TS diameter measured by magnetic resonance venography (MRV) and contrast T1-weighted magnetic resonance (contrast T1) were observed. To investigate these discrepancies, and considering that TS hypoplasia is associated with neurological disorders, we performed a post hoc analysis of prospectively collected data from 3 case–control studies on transient global amnesia (TGA), transient monocular blindness (TMB), and panic disorders while retaining the original inclusion and exclusion criteria. Magnetic resonance (MR) imaging of 131 subjects was reviewed to evaluate TS diameter and the location and degree of venous flow stenosis and obstruction.

MRV without contrast revealed that TS hypoplasia was observed in 69 subjects, whom we classified into 2 subgroups according to the concordance with contrast T1 observations: concordance indicated anatomically small TS (30 subjects), and discrepancy indicated that the MRV diagnosis is in fact flow-related and that TS is not anatomically small (39 subjects). The latter subgroup was associated with at least 1 site of venous compression/stenosis in the internal jugular vein (IJV) or the left brachiocephalic vein (BCV) (*P* < 0.001), which was significantly larger in patients than controls. Compensatory dilatation of contralateral TS diameter was only observed with MRV, not with contrast T1 imaging.

The clinical implication of these results is that using MRV only, IJV/BCV compression/stenosis may be misdiagnosed as TS hypoplasia. And contralateral TS have no compensatory dilatation in its diameter in contrast T1 imaging, just compensatory increased flow volume.

## INTRODUCTION

Anatomical asymmetry of transverse sinuses (TS) is a common finding and was considered a normal variant, as unilateral hypoplasia or aplasia is observed in 20% to 39% of cases.^1–3^ However, increasing evidence suggests that TS asymmetry is associated with, or can even negatively affect the clinical course of a number of neurological disorders.^4–7^ Adverse effects of TS hypoplasia include prolonged cerebral circulation time and impaired cerebral autoregulation linked to postcarotid-stenting hyperperfusion syndrome,^4^ and severe brain edema in middle cerebral artery infarction as a result of increased venous outflow resistance.^5^ Furthermore, TS hypoplasia is linked to the severity of high-altitude headache,^6^ and appears to correlate with white matter hyperintensity volume in patients with Parkinson disease.^7^ In all these studies, TS hypoplasia is considered not only as a normal variant but also a condition with potentially serious clinical implications on cerebral hemodynamic regulation. Of note is that these studies used either angiography or magnetic resonance venography (MRV) to evaluate TS flow, but none used contrast T1-weighted magnetic resonance imaging (contrast T1) to assess actual TS lumen shape and size.

**FIGURE 1:**
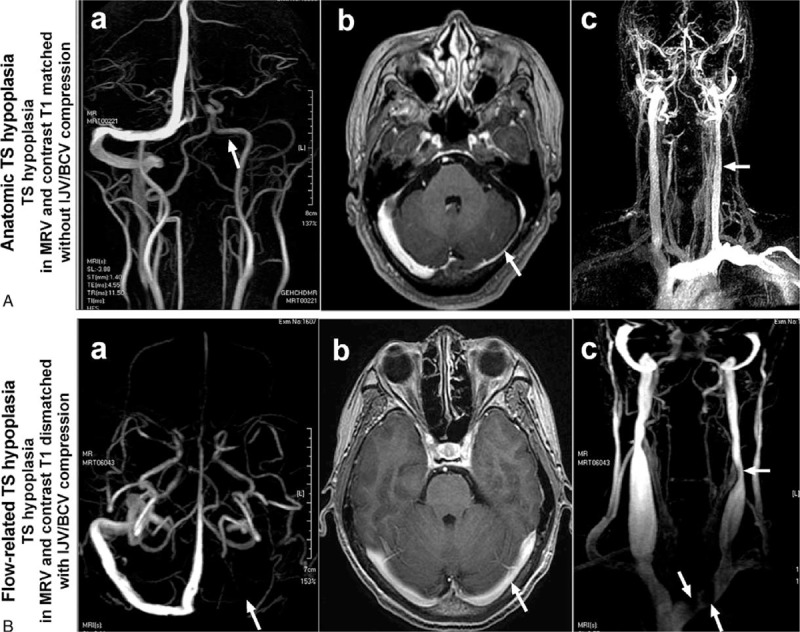
The concordance and discrepancy of transverse sinus (TS) in MRV and contrast T1-weighted magnetic resonance imaging (contrast T1). (A) Left TS in MRV (a, arrow) and contrast T1 (b, arrow) match in a study subject without IJV/BCV compression (c, arrow). (B) Left TS in MRV (a, arrow) and contrast T1 (b, arrow) mismatch in a study subject with IJV/BCV compression (c, arrows).

In our previous studies using MRV and contrast T1 to investigate transient global amnesia (TGA)^8^ and transient monocular blindness (TMB),^9^ we noted that there was not only a discrepancy in TS diameter measured by MRV and contrast T1, but also the discrepancy with correlating the internal jugular vein (IJV) stenosis/compression. This observation was consistent with another previous study.^10^ We found that some subjects displayed TS asymmetry on both MRV and contrast T1 (Figure 1A), which was deemed to be anatomical asymmetry. However, the others displayed TS asymmetry only on MRV, and not on contrast T1 (Figure 1B). We discovered that for subject with discrepant MRV and contrast T1, TS asymmetry was flow-related because of its association with IJV or left brachiocephalic vein (BCV) compression, as revealed by time-resolved imaging of contrast kinetics (TRICKS) of magnetic resonance (MR) imaging. In previous studies of TS hypoplasia using either angiography or MRV, a compensatory increase of contralateral TS diameter is commonly observed. To our knowledge, no one has evaluated the actual contralateral TS lumen using contrast T1. To evaluate the difference of TS hypoplasia observed between MRV and contrast T1, we retrospectively reviewed MR imaging from three prospective studies.^8,9,11^ This is the first study intending to clarify the morphology and hemodynamic characteristics of TS hypoplasia simultaneously and to explain the discrepancy of TS hypoplasia by different MR imaging.

## METHODS

### Subject Selection

We retrospectively reviewed the MR images of subjects enrolled from our previous prospective studies of venous outflow impairment in TGA,^8^ TMB,^9^ and panic disorder.^11^

The detailed research methods and population composition of the TGA, TMB, and panic disorder patients and their age- and gender-matched controls can be found in the original reports.^8,9,11^

TGA patients were prospectively recruited from the Neurology Department of Taipei Veterans General Hospital. All patients were examined by a neurologist, and TGA was diagnosed according to criteria modified and validated by Hodge and Warlow.^12^ Age- and gender-matched individuals for the control group were recruited prospectively from people receiving physical check-ups and who had no history of neurologic signs or symptoms.^8^

Patients diagnosed with TMB were recruited from consecutive outpatients of the Neurology Department of Taipei Veterans General Hospital and from referrals for cerebrovascular survey by ophthalmologists or other physicians. All patients were examined by 1 neurologist and were questioned about the characteristics of their transient loss of vision using a standardized questionnaire. The control group was selected from individuals receiving physical check-ups who had no carotid stenosis and no history of visual problems.^9^

For panic disorder, patients were consecutively referred by the psychological clinics of Taipei Veterans General Hospital (Dr. Hong) and were diagnosed according to the Diagnostic and Statistical Manual of Mental Disorders, 4th edition (DSM-IV-TR) criteria.^13^ Patients who were unwilling to participate or had history of hypertension, diabetes, smoking, stroke, ischemic heart disease, congestive heart disease, arrhythmia, pulmonary diseases, carotid or intracranial artery stenosis, and malignancies were not included.^11^

Overall, 131 patients were included in our study.

### MR Imaging Study

Examinations of all patients and controls were performed with contrast-enhanced MR imaging using a 1.5 T MR imaging scanner (GE Medical Systems, Milwaukee, WI).

The protocol included TRICKS, contrast-enhanced axial T1-weighted magnetic resonance imaging (contrast T1), and MRV. The MRV used was a phase contrast based contrast-free angiography image, acquired with Inhance 3D Velocity (a special software of GE Medical Systems). We used the following MR sequences: Inhance 3D Velocity MRV: sagittal plane repetition time (TR)/echo time (TE)/flip angle (FA): 11.7/4.5/10°, matrix size: 320 × 256, field of view (FOV): 24 cm × 21.6 cm, slice thickness: 1.4 mm with interpolation to 0.7 mm slice interval using a parallel imaging technique, acceleration factor: velocity encoding: 25 cm/s. TRICKS: coronal plane TR/TE/FA: 3.1/1.1/30°, matrix size: 320 × 192, FOV: 34 cm × 30.6 cm, slice thickness: 3.2 mm with interpolation to 0.8 mm slice interval. Contrast-enhanced T1 spoiled gradient recalled (SPGR) acquisition in the steady-state MR sequence: axial plane TR/TE/FA: 8.6/2.5/15°, matrix size: 320 × 256, FOV: 24 cm × 18 cm, slice thickness: 3 mm. Only those patients and controls who had complete studies with MR imaging were included in this study.

**FIGURE 2:**
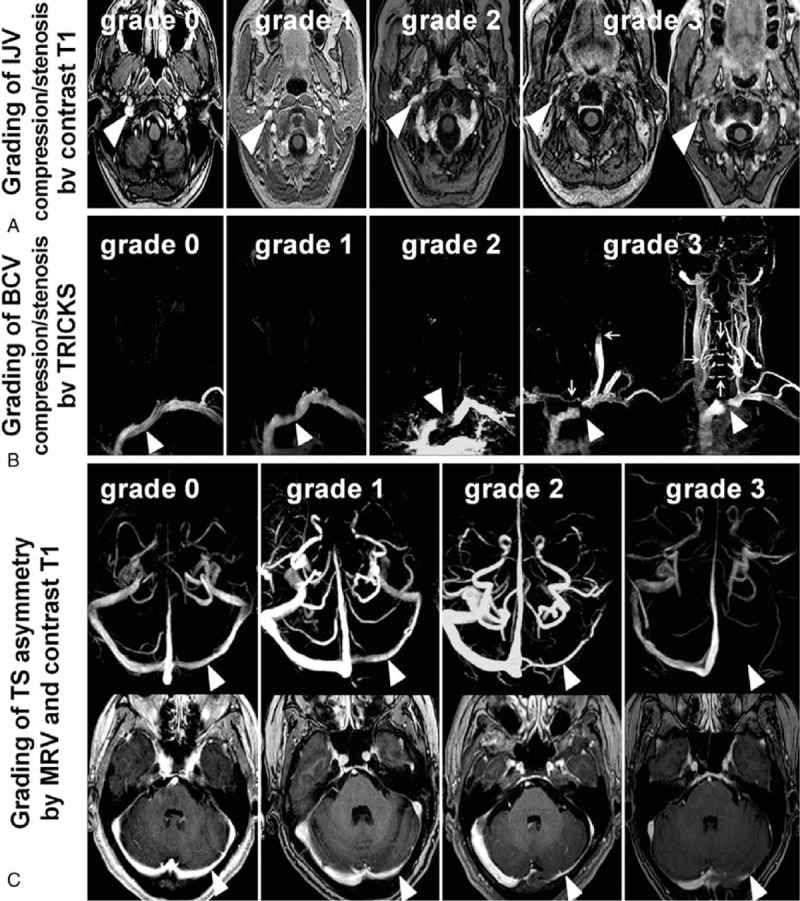
Grading of compression/stenosis using MR imaging. (A) Grading of IJV compression/stenosis by contrast T1: grade 0: normal round or ovoid; grade 1: mild flattening; grade 2: moderate flattening; grade 3: severe flattening, pinpoint or not visualized. (B) Grading of left BCV compression/stenosis by TRICKS: grade 0: normal (arrowhead); grade 1: BCV with mild filling defect by the aortic compression (arrowhead); grade 2: left BCV interrupted at the aortic arch (arrowhead) with filling defect, but without collateral; grade 3: left BCV compression/occlusion (arrowhead) with different types of venous collaterals filling and reflux: venous flow drains across the midline into the right IJV through the anterior cervical veins from left subclavian vein (vertical arrow); IJV reflux (horizontal arrow), the contrast medium injected from the left subclavian vein appears retrograde into the left IJV; Presence of collaterals of vertebral venous system, from the left subclavian vein draining directly through intrarachidian anastomoses to contralateral side at different levels (arrows). (C) Grading of TS asymmetry by MRV: grade 0: symmetrical TS; grade 1: TS asymmetry ≤50%; grade 2: TS asymmetry >50%; and grade 3: aplasia or signal absent (arrowhead pointing out locations for comparison). Grading of TS asymmetry by contrast T1: grade 0: symmetrical TS; grade 1: TS asymmetry ≤50%; grade 2: TS asymmetry >50%; grade 3: aplasia or signal absent (arrowhead pointing out locations for comparison).

IJV morphology was assessed at the upper IJV (at C1–2 level), middle IJV (at C3–5 level), and lower IJV (at C6–T2), using contrast T1. IJV compression/stenosis was evaluated according to the following criteria by Zaharchuk et al^10^ grade 0 = normal round or ovoid appearance; grade 1 = mild flattening; grade 2 = moderate flattening; grade 3 = severe flattening or not visualized (Figure 2A).

Left BCV obstruction was graded according to the filling defect shown on TRICKS as follows: grade 0 = normal, or compression ≤20%; grade 1 = compression >20% and ≤80%; grade 2 = compression >80%; grade 3 = grade 2 plus presence of different types of venous collaterals^8^ (Figure 2B). The vertebral/intraspinal/neck collaterals were evaluated using the TRICKS images, focusing on the presence of posterior condylar veins and collaterals of the vertebral venous system (Figure 2B).

TS diameter on MRV and contrast T1 was measured in cm at the mid-lateral portion of the TS using the method by Fofi et al,^1^ as we found that this location of the TS could be easily identified and measured with certainty in almost all cases. TS morphology was graded using MRV (Figure 2C, top) and contrast T1 (Figure 2C, bottom), modified from the following criteria by Fofi et al^1^: grade 0 = TS symmetry or TS asymmetry ≤10% compared with the contralateral TS; grade 1 = TS asymmetry >10% and ≤50% compared with the contralateral TS; grade 2 = TS asymmetry >50% compared with the contralateral TS; grade 3 = aplasia, or TS signal absent. TS hypoplasia was defined as the indexed TS being >50% of the contralateral TS, including grade 2 or 3.

1 neuroradiologist and 1 neurologist reexamined all MR images. Both physicians were well trained in reading neuroimaging and were blinded to the subjects’ clinical characteristics. A consensus meeting was conducted to discuss any problem or disagreement. The intraclass correlation coefficient for grading was used to assess interrater agreement for IJV morphology, BCV obstruction, and TS asymmetry assessments. Interrater reliability was larger than 0.91 for all 3 assessments.

The study was approved by the Institutional Review Board of Taipei Veterans General Hospital.

### Statistical Analysis

TS size measurements were presented as median and interquartile range (IQR). We compared the frequency of TS hypoplasia among different groups of patients and controls using Fisher exact test or a Chi-squared test, and conducted post hoc analysis of pairwise comparisons using the Bonferroni correction. Continuous data were compared using the nonparametric Kruskal–Wallis test, and pairwise comparisons were conducted using the Wilcoxon rank-sum test with the Bonferroni correction. Two-sided *P* values of less than 0.05 were considered to indicate statistical significance. All analyses were performed with SAS 9.2 (SAS Institute, Cary, NC).

## RESULTS

A total of 131 subjects (90 patients and 41 controls, 70 men and 61 women, mean age 54.0 ± 15.1 years, range: 17–86 years) were enrolled in our studies of TGA, TMB, and panic disorders during the years 2008 to 2012.

The distribution of control subjects and patients with TS hypoplasia and different clinical disorders is presented in Table 1. Out of the 131 study subjects, 69 had TS hypoplasia according to the MRV criteria, 65 on the left- and 4 on the right-hand side. Out of these 69 subjects, 30 were confirmed using contrast T1, which means that the TS was indeed small anatomically (defined as Anatomic TS hypoplasia), and 39 were not confirmed using by contrast T1, which indicates that TS was not small anatomically but that venous flow may be lower or slower (defined as Flow-related TS hypoplasia). TS hypoplasia frequency was different among the patient and control groups (*P* = 0.005) and a post hoc analysis showed a significant difference in frequency between TGA patients and controls (*P* = 0.001) and between TMB patients and controls (*P* < 0.001) (Table 1).

**TABLE 1:**
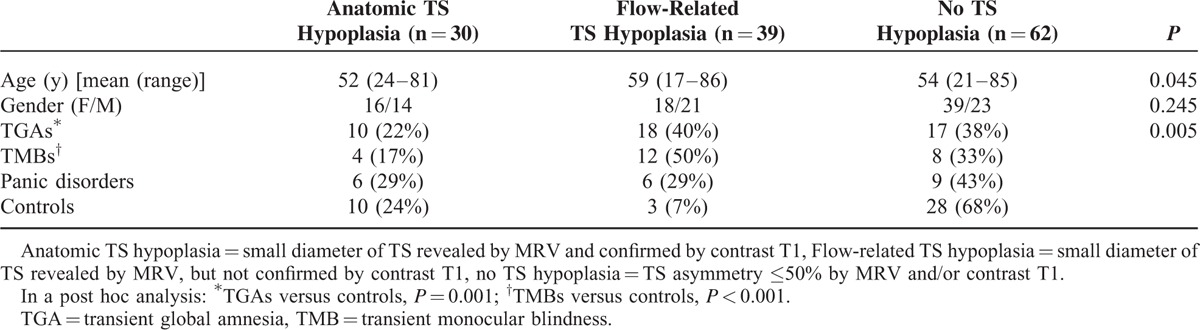
Transverse Sinus (TS) Hypoplasia in Different Groups of Patients and Controls Based on MR Imaging

Frequency of IJV and left BCV compression in the 2 types of TS hypoplasia and controls, based on MR imaging, is shown in Table 2. In a post hoc analysis, Flow-related TS hypoplasia was found to be significantly more frequently associated with BCV compression than No TS hypoplasia (*P* = 0.013) and with at least 1 site of venous compression/stenosis in the IJV or BCV (Flow-related TS hypoplasia vs. No TS hypoplasia, *P* < 0.001; Flow-related TS hypoplasia vs. Anatomic TS hypoplasia, *P* = 0.004) (Table 2 and Figure 1). However, none of these differences were found between Anatomic TS hypoplasia and No TS hypoplasia. Of note, as only 2 patients had stenosis at lower level IJV (at C6–T2) in our sample, we did not analyze the data for lower IJV stenosis.

**TABLE 2:**
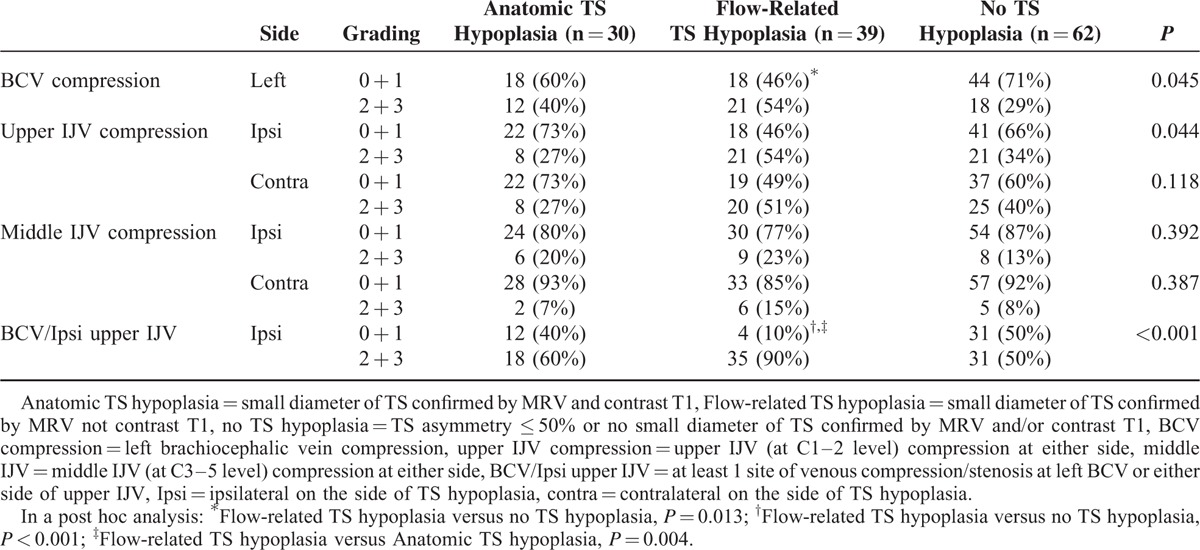
Frequency of Internal Jugular Vein (IJV) and Left Brachiocephalic Vein (BCV) Compression in Different Types of Transverse Sinus (TS) Hypoplasia and Controls Based on MR Imaging

**FIGURE 3:**
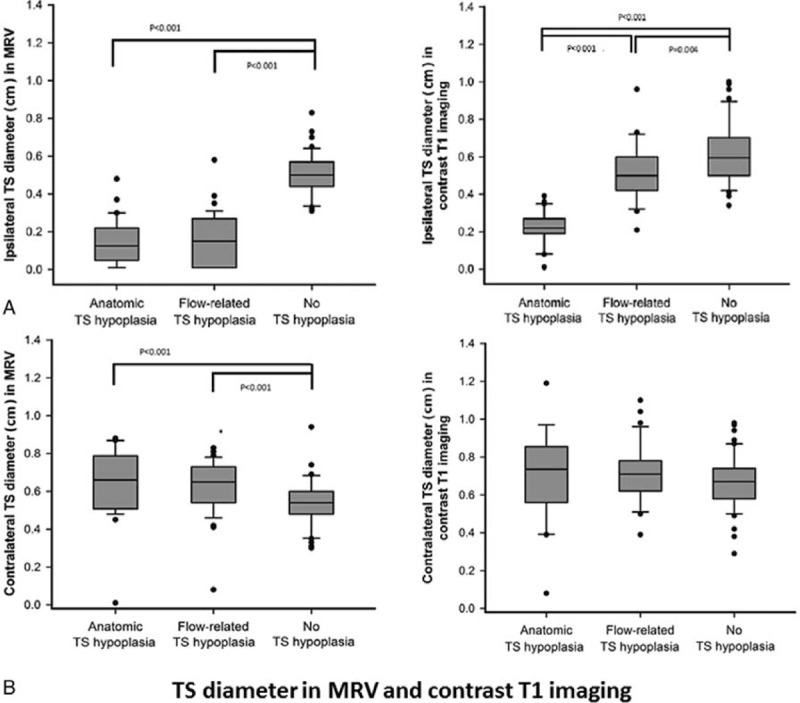
Comparison of bilateral transverse sinus (TS) using MR imaging. (A) Left panel: Ipsilateral TS diameter in MRV. Right panel: Ipsilateral TS diameter in contrast T1. (B) Left panel: Contralateral TS diameter in MRV. Right panel: Contralateral TS diameter in contrast T1. Data are presented as median (interquartile range), and 10% to 90% of study subjects were included between the upper and lower bars.

For ipsilateral TS hypoplasia, using MRV imaging (Figure 3A, left panel), TS diameter median values in cm [median (IQR)] were 0.12 (0.06–0.22) for Anatomical TS hypoplasia and 0.15 (0.01–0.27) for Flow-related TS hypoplasia. Both were significantly smaller than their control counterpart [0.50 (0.44–0.57)] (*P* < 0.001 in both cases) (Figure 3A, left panel). Using contrast T1 images (Figure 3A, right panel), TS diameter median values were 0.22 (0.19–0.27) for Anatomical TS hypoplasia and 0.50 (0.42–0.60) for Flow-related, again both were significantly smaller than their control counterpart [0.60 (0.50–0.70)] (Anatomical TS hypoplasia vs. control, *P* < 0.001; and Flow-related TS hypoplasia vs. control, *P* = 0.004, respectively). Though there was no significant difference in TS diameter between the 2 types of hypoplasia in MRV imaging (Figure 3A, left panel), the TS diameter of Anatomic TS hypoplasia was significantly smaller than that of Flow-related TS hypoplasia in contrast T1 imaging [0.22 (0.19–0.27) vs. 0.50 (0.42–0.60), *P* < 0.001] (Figure 3A, right panel).

For contralateral TS diameters, using MRV imaging (Figure 3B, left panel), TS diameter median values in cm [median (IQR)] were 0.66 (0.51–0.78) for Anatomical TS hypoplasia and 0.65 (0.54–0.73) for Flow-related TS hypoplasia. Both were compensatory larger than their control counterpart [0.54 (0.48–0.60)] (*P* < 0.001 in both cases). Using contrast T1 images (Figure 3B, right panel), the diameters were comparable in all 3 groups: Anatomical TS hypoplasia vs. Flow-related TS hypoplasia vs. control counterpart of No hypoplasia [0.74 (0.56–0.85) vs. 0.71 (0.62–0.78) vs. 0.67 (0.58–0.74), Chi-square = 3.83, *P* = 0.147] (i.e., no compensatory dilatation was observed).

## DISCUSSION

### Main Finding: Subgroups of TS Hypoplasia

In this MR imaging study, we found that TS hypoplasia revealed by MRV is not always congenital or anatomical. Half of TS hypoplasia diagnosed by MRV could not be confirmed by contrast T1, meaning that TS was not structurally small (Table 1). As contrast T1 is considered the criterion-standard measurement of TS lumen, TS hypoplasia was classified into 2 subgroups according to the concordance, or discrepancy, in the diagnosis of TS hypoplasia between MRV and contrast T1 observations. Concordant diagnoses (i.e., confirmed by contrast T1) indicate that TS is anatomically small, and were labeled Anatomical TS hypoplasia. Discrepant diagnoses (i.e., not confirmed by contrast T1) indicate that the MRV observation was not anatomical, but related to slow or low venous outflow. This was substantiated in our data by an association with BCV compression and with at least 1 site of venous compression/stenosis in the IJV or BCV. This was not the case for both Anatomical TS hypoplasia and No TS hypoplasia groups (Table 2). For this reason, discrepant diagnoses were labeled Flow-related TS hypoplasia.

### Clinical Implications of the Existence of TS Hypoplasia Subgroups

In previous studies,^8,9^ we demonstrated that IJV or BCV compression is linked to TGA or TMB, and found the frequency of TS hypoplasia in those patients to be significantly higher in MRV, without being the case in contrast T1. Consistent with this observation, the present study found Flow-related TS hypoplasia to be much more frequent in all 3 patient groups (TGA, TMB, panic disorder) than in controls (Table 1). The discrepancy between MRV and contrast T1 may indicate that Flow-related TS hypoplasia might be more clinically relevant to these neurological disorders than Anatomical TS hypoplasia. As described above, increasing evidence suggests that many neurological disorders are linked to TS hypoplasia or venous outflow obstruction.^4–9,11^ If we know that TS hypoplasia is secondary to venous obstruction, we might have some ways of intervening and solve the venous outflow obstruction.^14,15^ In the case of anatomical variation, the method of management might be different.

However, we stress that extrapolating these findings to other clinical disorders without evidence should be avoided. Because our study population consists of patients with TGA, TMB, and panic disorder, it is associated with a high prevalence of venous outflow obstruction. As a consequence, the frequency of TS hypoplasia may be different in other diseases without venous outflow obstruction.

Furthermore, in those studies that reported various diseases associated with TS hypoplasia^4–7^ such as Parkinson disease, high altitude headache, postcarotid-stenting hyperperfusion syndrome, and severe brain edema in middle cerebral artery infarction, the 2 subtypes of TS hypoplasia were not separated and it is not possible to know which type of TS hypoplasia is really related to, or has a great impact on, specific neurological disorders. Further studies are needed to distinguish which of the 2 TS hypoplasia subtypes is associated with clinical disorders including prolonged circulation time or impaired cerebral autoregulation, and to evaluate the different clinical implications of TS hypoplasia subtypes.

### Contralateral Compensation in TS Hypoplasia

Looking at contrast T1, we made an interesting observation: the contralateral TS diameter was not compensatory enlarged, which is in disagreement with the common belief of compensatory TS dilatation. The explanation is that apparent contralateral TS diameter dilatation in MRV is instead due to a compensatory increase in the flow volume.

### MR Imaging and TS Lumen

MRV without contrast is based on venous blood flow, and may be influenced by venous flow artifacts such as flow velocity and flow amount. Therefore, it is not suited to represent TS diameter accurately. Contrast T1 is derived from contrast-enhanced techniques by intravenously injecting a contrast agent into the vascular system. Considering that contrast-enhancing techniques are relatively insensitive to signal loss, they provide high quality images with fewer artifacts than noncontrast-enhanced MRV. Thus, flow artifacts will be maximally reduced in contrast-enhanced T1.^16^ Moreover, the contrast T1 applied in this study was SPGR acquisition in the steady-state MR sequence. This sequence can provide better soft tissue contrast than others to delineate the TS and jugular venous system anatomical structures. In conclusion, the other benefits of this contrast-enhanced T1 weighted imaging SPGR is better than T2 weighted imaging in delineating the anatomy because of lessened motion artifacts by shorter acquisition time, and because the injected contrast SPGR can enhance the venous lumen and eliminate venous flow artifacts.^17^ In our study, the wall of the venous system was clearly shown by the contrast-enhanced T1 weighted imaging SPGR. Therefore, we believe contrast T1 is reliable to estimate TS lumen diameter. In summary, according to the basic principle of MR imaging, TS diameter measured in this study using contrast T1 is reliably representing actual TS lumen diameter, while the Inhance 3D velocity MRV^18^ represents venous flow velocity and flow amount.

### Limitations

While we believe in the accuracy of our conclusions and soundness of our methods, the following limitations of our study should be considered. Firstly, due to the difficulty to measure TS area size when TS is not elliptical or circular, which happened regularly, only TS diameter was used. Secondly, the reliability of contrast T1 to measure TS lumen diameter may be questioned. However, considering that contrast-enhancing techniques are relatively insensitive to signal loss and that they remove most flow artifacts, we believe that contrast T1 is reliable to estimate TS lumen diameter. Thirdly, data from MR imaging were collected prospectively but were reviewed retrospectively. Consequently, our results may be subject to biases inherent to retrospective studies.^19^ However, our study data were prospectively collected and we think that this analysis enabled us to understand the existence of 2 types of TS hypoplasia, with 1 type associated with venous outflow obstruction. The results from this exploratory study may generate specific hypotheses, which can then be tested in specifically designed studies in the future. Further studies are needed to clarify the hemodynamic changes of each subtype of TS hypoplasia.

## CONCLUSIONS

In conclusion, TS hypoplasia can be divided into 2 subgroups: Anatomical TS hypoplasia and Flow-related TS hypoplasia, as defined by the discrepancy between MRV and contrast T1. Flow-related TS hypoplasia was found to be associated with some neurological disorders of venous outflow impairment, such as TGA and TMB. Further studies are needed to clarify the clinical implications of each subtype of TS hypoplasia, using ultrasound of IJV and vertebral vein (VV). Using contrast T1, we also found no evidence for compensatory dilatation of contralateral TS diameter, which indicates that it is flow volume, and not TS diameter, that is compensatory increased.
